# Automated Execution of Data Pipelines based on Configuration Files.

**DOI:** 10.12688/openreseurope.21019.1

**Published:** 2025-09-19

**Authors:** Károly Bósa, Paul Heinzlreiter

**Affiliations:** 1 RISC Software GmbH, Hagenberg, 4232, Austria

**Keywords:** data preprocessing, data integration, data pipelines, configuration, automation.

## Abstract

**Background:**

Data preparation is a fundamental aspect of data engineering, a prerequisite for later tasks such as data visualization, reporting, and training machine learning models. Despite the recurring patterns in data transformation processes, the specific steps often vary depending on the project context, data sources, and application domain.

**Methods:**

To address these challenges, this paper presents a flexible and extensible framework that enables the coordinated execution of modular data processing steps defined in a configuration file. By adopting a declarative, configuration-driven approach, the framework promotes modular, step-by-step development while substantially improving code reuse, maintainability, and adaptability. The framework also supports basic iterative execution constructs, such as loops and limited recursion, within the data pipeline definitions to accommodate more complex workflows.

**Results:**

By enabling the reuse of existing code snippets, the framework shifts development efforts toward enhancing and refining a shared code base, rather than repeatedly creating project-specific, disposable implementations. The long-term benefits of this approach become increasingly apparent as the system evolves. As more generalized modules and functions are developed, they can reduce duplication and improve maintainability without sacrificing flexibility.

**Conclusions:**

To assess the effectiveness of the framework, we apply cyclomatic complexity as a metric, demonstrating how the proposed approach impacts the development effort across some relatively simple, real-world data engineering scenarios.

## Plain language summary

This paper presents a flexible software tool that helps to organize and automate data preparation steps for e.g. reports, visualizations or machine learning. Instead of writing individual program code for each project, users can easily link different data processing steps as building blocks by listing them in a configuration file. This makes it easier to reuse, update and adapt the process to different needs as well as saves time and reduces repetitive work. The tool also encourages the creation of a shared library of reusable processing parts.

## 1. Introduction

Data preparation tasks like validation, cleaning, or data integration are crucial to support data science and machine learning. At their core they consist of multiple distinct stages like retrieving the data from the source (Extract), transforming the data in some way (Transform) and loading it into its target data store or storing it in a specific target file format (Load). Such an ETL process [ Vassiliadis *et al.*, 2002] can be modified by changing the ordering of its stages, for example turning it into an ELT process, if the raw data is first loaded into a cloud-based data processing and storage system, and then being transformed there, making use of the computational capacities on the cloud.

To streamline our ETL processes, we recently switched our data preparation implementations to Python [ Van Rossum & Drake, 2009] due to the strong library support and reduction of boilerplate code. This includes building most of the data transformations on top of the Pandas [ McKinney, 2010] library as well as using Dask [ Rocklin, 2015] for the data-parallel execution of jobs with larger input datasets. Relying on these libraries and using Python as the main implementation language reduces the implementation effort and amount of code duplication significantly, since most of the functionality required is already provided by the Pandas library.

However, across a set of diverse use-cases coming up over time, we noticed that a lot of effort is still spent on repeated implementation of similar code snippets, violating the DRY (don’t repeat yourself) principle [ Hunt & Thomas, 1999]. This typically includes topics like

- Data access to local and remote files as well as databases
- Data validation and transformations like resampling, data cleaning, fixing timestamps and related basic tasks.

Building on top of Python and Pandas these tasks typically only require small code segments to implement; it is mostly a thin wrapper over the already existing library. But even this small amount of code requires implementation, testing, code review, source code management, deployment and all related steps of a full software development process if done properly. All this effort adds up, it feels repetitive for experienced developers while still introducing a learning curve for developers who are not familiar with Python and its libraries.

### Benefits of code reuse

To prevent the re-implementation of these code snippets we investigated whether we could move the variable parts to configuration files, which is anyways a good software engineering practice. key steps involved in ETL processes and data preparation for Machine Learning (ML) and Artificial Intelligence (AI) tasks in general are similar enough that the implementation can be reused. If code gets reused it can be extended and refined over time, for example making it more resilient towards errors in the input data. On the other hand, reimplementing similar code reintroduces errors [ Wagner *et al.*, 2016], consequently increasing the effort. By reusing existing code snippets, effort can be invested into improving an already existing code base as opposed to developing new “single use” code. This challenge is particularly evident in data preparation tasks, where datasets often undergo exploratory analysis and may ultimately be discarded if they fail to meet certain quality criteria.

### Minimalist Configurable Pipeline (MCP) Framework

Our projects mostly require simple data pipelines, where typically a limited amount of data is being read from a data source – a database, a structured text or binary file – transformed and stored back to a data sink such as a database or a Parquet [ Vohra, 2016] file. While the transformation step is often specific to the use-case, reading and storing data is a general task required in nearly all our data preparation codes. This consequently led to code repetition specifically around data access. Other common areas of code repetition are, for example resampling of time series data and data merging.

The commonalities around data access were identified as easily reusable, such as access to databases or abstracting away the type of file storage getting accessed, whether it is a local file, a cloud bucket, or a network file share like a Microsoft Windows or Teams share. We have implemented these common tasks multiple times before, with only slight variations in functionality, across different use cases.

Based on this observation, we focused on the development of a methodology, which is based on three key pillars:

- **Minimalist Configurable Pipeline (MCP) Framework [ MCP, 2025]:** Our primary contribution reported in this paper is the development of the MCP, a flexible pipeline framework driven by a configuration file. This allows for dynamic execution of data pipelines without modifying code.
- **Reusable Source Code and Best Practices**: Recognizing that a configuration framework alone is not enough to address the challenges we face, our team collaboratively established a set of recommendations for creating use case-independent general re-useable source code and lay down a simple set of rules that allows us to exploit the potential of the MCP framework, see Section 4.
- **Implementation of use case independent Library**: Finally, to bring our vision into practice, we implemented several algorithms and began developing a library that consolidates routines that are independent of specific use cases. These routines are designed to be easily reusable for addressing future problems. While the initial phase involved a learning curve and required some extra effort and resources, the investment is now starting to yield benefits, as discussed in Section 5.

Using a configuration file in YAML format, MCP moves us from modifying code to modifying configurations. The data access and transformations to be performed are read from the YAML configuration file and executed, while the actual python code remains unmodified.

### MCP key features and design goals

We place strong emphasis on the practical applicability of the MCP framework. It is designed to integrate seamlessly with existing codebases, enabling reuse of existing solutions and minimizing the need to reimplement mundane boilerplate code.

Data access is defined declaratively within configuration files. This includes the familiar practice of specifying database connections, but also extends to more flexible concepts, such as invoking functions from external modules. A key feature of the framework is its composability: an MCP data pipeline is structured as a series of simple ETL transformation stages. These stages, which are organized as child pipelines, are composed of bundles containing statement references. Each reference may point to (Python) functions in external modules or to other bundles. It is also possible to iteratively execute referenced components on some data structure using loop constructs, enabling the creation of hierarchical execution chains that originate from a single entry-point.

Equally important is to ensure that the configuration files remain simple and intuitive. Introducing excessive complexity at the configuration level would undermine the framework’s purpose, especially considering that Python already offers a concise and expressive syntax that naturally reduces boilerplate.

The rest of the paper is structured as follows: Section 2 reviews the state of the art in the field. Section 3 provides a brief overview of data pipelines within the MCP framework and explains their functionality. Section 4 outlines the requirements that Python source code must meet to serve as a building block in MCP data pipelines. It also highlights the features offered by the framework and presents some best practices for effective implementation.

Section 5 showcases some real-life use cases that have been successfully re-implemented within the MCP framework. Additionally, it evaluates these implementations using the software metric cyclomatic complexity [ McCabe, 1976] to validate the practicality and effectiveness of our data pipeline solutions. Finally, Section 6 concludes the paper.

## 2. Related work

The issue of code duplication and “reinventing the wheel” is well-known in software development. Several approaches like modularization and object-oriented programming have been developed to address it. Additional ideas include, for example, low-code or no-code approaches and graphical programming, which have been moderately successful in limited application domains.

### Data validation, cleaning and merging

In the domain of data integration and preparation including *extract-transform-load (ETL)* processes, data is typically transferred from a source to a sink, both of which can take various forms such as databases, structured binary files, text files, or network streams. Logically, these processes can be decomposed into multiple stages, ideally connected by well-defined interfaces. These interfaces promote modularity and interchangeability and can be used either within a single process or across process boundaries.

Several solutions have emerged based on this modular concept, distinguishable primarily by their level of abstraction. A key distinction can be drawn between high-level orchestration and workflow scheduling tools (e.g., *Apache Airflow* [ Airflow, 2024], *Prefect* [ Prefect, 2024], *Dagster* [ Dagster, 2024]), mid-level tools such as *Flyte* [ Flyte, 2024] and *Dask* [ Rocklin, 2015], and lower-level solutions like *DBT (Data Build Tool)* [ DBT, 2024] and various command-line utilities.

One of the most prominent high-level orchestration tools is Apache Airflow, an open-source platform for designing, scheduling, and monitoring complex workflows. Airflow allows users to define workflows as *Directed Acyclic Graphs (DAGs)*, where each task represents a discrete unit of work, and dependencies define execution order. It provides robust capabilities for scheduling, dependency management, and workflow monitoring. Airflow integrates seamlessly with a wide range of systems and services, making it well-suited for orchestrating complex data pipelines and automation tasks. Thanks to its extensibility and scalability, Apache Airflow is widely adopted across domains such as data engineering, data science, and DevOps for managing large-scale workflows.

Our proposed approach is not intended to compete with high-level orchestration tools like Apache Airflow or mid-level frameworks. Instead, we position the MCP framework as a low-level tool, comparable to DBT (Data Build Tool), but with a different focus.

DBT is a widely adopted tool that concentrates on the Transform stage of the ETL process. It uses batch processing to perform transformations by reading from and writing to databases. It allows users to express transformations using SQL [ Groff & Weinberg, 1999], making it approachable for analysts and engineers familiar with database technologies. However, despite its strengths, DBT comes with notable limitations:

- **Limited Python Integration**: While DBT supports Python models, the functionality is constrained. There are performance limitations and a lack of native support for advanced machine learning libraries such as *scikit-learn* or *TensorFlow*, reducing its flexibility for more sophisticated use cases.
- **Restrictions in DataFrame APIs**: DBT’s Python models do not fully support key features from *Pandas*, such as advanced I/O operations or custom extensions. Platform compatibility, debugging complexity, and runtime performance further add to the friction. These issues are amplified by DBT's “SQL-first” design, where Python models lack the same level of optimization, integration, and native support as SQL models.
- **Limited Output Flexibility**: DBT is tailored for in-database transformations, typically producing tables or views within the same platform. It offers minimal native support for exporting results to file-based formats such as *Parquet*, *CSV*, or *JSON*, which can be a constraint in hybrid workflows.
- **No Field-Level Lineage**: DBT provides strong support for table-level dependency management but lacks field-level lineage. This can be a limitation for more granular tracking of how individual columns are transformed across models. This limitation may lead to challenges in debugging, impact analysis, and auditing, particularly in large or complex data pipelines.
- **Readability Challenges**: While DBT makes writing SQL easy, maintaining it can become difficult on a scale. Large projects often result in complex SQL transformations that are hard to understand and modify, sometimes encouraging the creation of redundant tables instead of reusing or updating existing ones.

In contrast, the MCP framework focuses on accelerating the development of data pipelines through code reuse via configuration. The core idea is to keep the transformation logic in the native programming language (e.g., Python), with full access to its ecosystem including libraries for data science, machine learning, and complex logic.

Unlike tools such as Airflow, which are geared toward orchestrating and scheduling workflows, MCP does not aim to manage high-level pipeline execution. Instead, it complements these systems by offering a lightweight, modular, and reusable way to define transformation logic. Managing such fine-grained logic using Airflow’s DAGs often results in unnecessary complexity and overhead, something MCP aims to simplify.

### Low code approaches for data pipelines

Several low-code solutions exist for configuring data pipelines through graphical interfaces *. Apache NiFi* [ Nifi, 2024], for example, provides a broad range of connectors for data sources, sinks, and filters, and is designed to be horizontally scalable to handle large volumes of data. NiFi emphasizes a data streaming paradigm, where data continuously flows from source to sink in near real-time. A key advantage of NiFi is its graphical user interface, which allows users to build and manage pipelines visually via a web-based dashboard. This is especially useful for users with limited programming experience.

However, despite its user-friendly interface, NiFi comes with a steep learning curve. As a powerful, enterprise-grade tool, it offers extensive functionality that can be overwhelming for beginners and requires significant effort to use effectively at scale. A similar low-code approach is offered by *Pentaho Data Integration* [ Pentaho, 2024], which also supports graphical pipeline configuration through an intuitive interface.

In contrast, the MCP framework targets developers who prefer or are already familiar with code-based development, particularly in Python. Instead of providing a visual interface, MCP focuses on reducing the effort involved in implementing data pipelines in code, streamlining the process through modular configuration. This approach offers a lower learning curve for Python users and promotes adherence to software engineering best practices, such as version control, unit testing, and modular design.

Tools like NiFi do offer some versioning capabilities via XML-based pipeline exports and a workflow registry. Due to their auto-generated output these tools are ill-suited for version control systems typically used in software development and *infrastructure-as-code* deployments. As a result, understanding and tracking changes in such files can be cumbersome and opaque unlike traditional source code, which is easier to manage, review, and collaborate on.

### Configuration file formats and merging

Another relevant area of the state of the art involves the management of file-based configurations, including the associated tooling and file formats *. YAML* [ Yaml, 2021] is a well-established and widely adopted configuration language. It is commonly used to describe infrastructure and automation tasks, such as Kubernetes container orchestration and CI/CD pipeline definitions.

In the Python ecosystem, *Gin* [ Holtmann-Rice *et al*., 2018] is a popular configuration management library. It uses familiar Python-like syntax and supports hierarchical configuration files, making it particularly effective for managing shared hyperparameters and complex nested configurations. However, Gin lacks built-in capabilities for dynamic composition, limiting its flexibility when working with multiple or evolving configuration sources.

To address these limitations, several tools have been developed to enable the merging of configurations from various sources. *OmegaConf* [ Yadan *et al.*, 2019] provides mechanisms for merging YAML files, while *Hydra* [ Yadan, 2019] extends OmegaConf with powerful features like command-line overrides and job launching with multiple configuration variants.

The MCP framework can be viewed as a natural extension of these approaches. While it relies on YAML as a versatile and human-readable configuration format, MCP goes further by enabling the definition of data pipelines composed of Python functions and special control structures, such as loops. Importantly, MCP supports the dynamic composition of YAML-based configurations, such as combining multiple files or applying overrides, through its integration with the Lasagna tool [ RISC\Lasagna, 2021]. Developed internally within our organization, Lasagna addresses YAML merging in a way that is lightweight and significantly simpler than OmegaConf or Hydra.

This capability makes it easy to adapt existing pipeline configurations to new use cases with minimal effort, which promotes reuse, modularity, and maintainability in real-world data pipeline development.

### Contribution of MCP

The primary contribution of the MCP framework is its lightweight integration with standard Python programming, without the need to install or learn a separate toolkit, as required by many of the alternatives mentioned above. MCP streamlines code reuse, helping developers avoid repetitive boilerplate code and reducing the likelihood of introducing errors through redundant implementations. By making reuse easier and more intuitive, MCP encourages developers to focus their efforts on writing clean, modular, and reusable code, rather than redundantly redeveloping the same functionality for slightly different use cases.

## 3. Short overview and methods

The primary goal of the framework is to eliminate the need to reimplement common steps in typical ETL processes, especially when only minor variations from existing workflows are required. It is designed to aim to address a wide range of recurring tasks, including:

- Connecting to and accessing various types of databases
- Reading from and writing to widely used file formats, such as CSV, compressed archives, and binary formats like Microsoft Excel
- Interacting with remote file storage systems, including Amazon S3, Windows network shares, and Microsoft Teams folders
- Traversing directory structures to perform operations on individual files within a logical loop, which is particularly useful for processing data organized in hierarchical file systems
- Performing data type conversions
- Applying commonly used data manipulation and transformation operations

The system allows data pipelines to be defined declaratively through a configuration file, which references the implementations of the individual pipeline stages. Each stage is represented by a function that is dynamically loaded either from Python source files or from dynamic libraries written in other programming languages, such as C#. For example, integration with C# can be achieved using a wrapper code that leverages *Python.NET* [ PythonDotNet, 2024] to interact with *the .NET Common Language Runtime (CLR).*

The configuration file specifies the sequence of ETL steps by linking each step to its corresponding implementation. The MCP runtime module processes this configuration, constructs the execution plan by resolving the order of operations, and then orchestrates the pipeline’s execution accordingly.

### Configuring the data pipeline in YAML format

In the MCP framework, a YAML configuration file typically consists of key-value pairs, like other structured configuration files. These pairs may define both predefined parameters, such as input data locations, output directories, or database settings and user-defined constants, grouped under the sections like *input_path, output_path, tmp_paths, database_configs* and *further_configuration*.

Beyond these parameters, the configuration file includes three key structural elements *: imports, pipelines*, and *entry_point*. The section introduced by the *imports* keyword lists all modules that should be dynamically imported at runtime. These modules must contain the functions that are referenced later in the data pipeline definitions.

The *pipelines* section may include one or more pipeline definitions, each identified by a unique label. A pipeline is composed of a sequence of steps, which can reference Python functions, other pipelines, or special control-flow constructs such as *loop*. As detailed in Section 4, any function within the framework can register a list for iteration. When a *loop* statement is encountered, it retrieves the next registered list (of which there may be multiple) and iteratively executes the specified function or referenced pipeline for each element in the list.

The key word *entry_point* refers to the label of a pipeline defined in the *pipelines* section. This serves as the starting point for the program’s execution.

**Figure 1.**
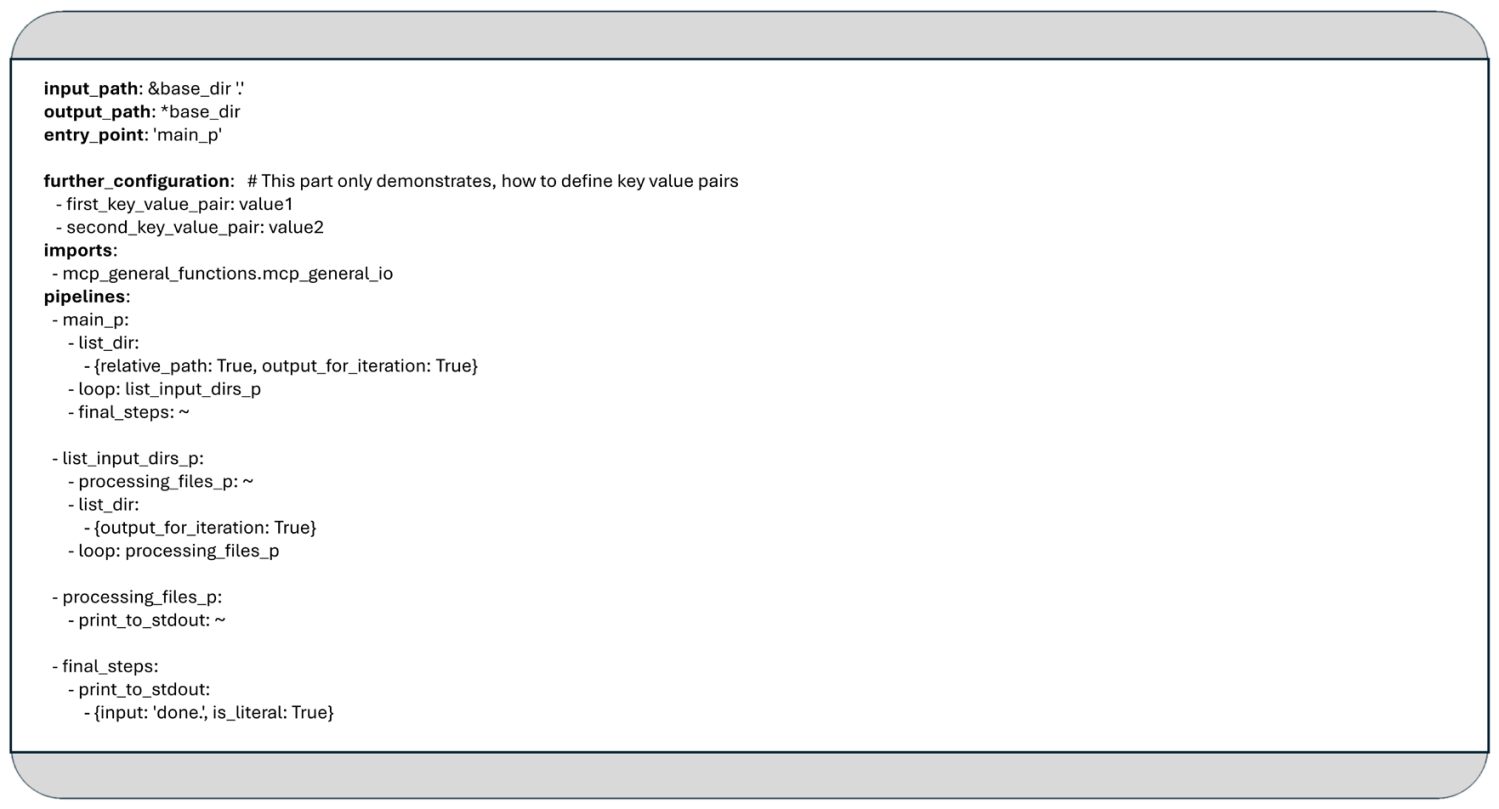
Simple YAML configuration to list the content of the given input folder in two levels deep.

Figure 1 shows an example YAML configuration that lists the contents of a given input directory and prints them on the screen. The main loop iterates over each item in the directory. If an item is a folder, the system generates a new list containing that folder’s contents. Inner loops then iterate through each subdirectory, printing its contents as well.

In MCP, a YAML-based data pipeline description typically consists of a set of small transformation bundles or (child-) pipelines, which can reference one another, call Python functions, or initiate loop constructs. The pipeline definition shown in Figure 1 is composed of the following key elements:

- It defines three child pipelines: "main_p", "list_subdirs_p", and "processing_files_p".
- It references two Python functions *, list_dir* and *print_to_stdout*, which are implemented in the module *mcp_general_functions.mcp_general_io*. These functions follow the coding guidelines and best practices outlined in Section 4.
- It includes two nested loops: the outer loop iteratively calls the "list_subdirs_p" pipeline, while the inner loop does the same with "processing_files_p".

As seen in Figure 1, some pipeline elements contain the tilde character (~), which acts as a placeholder when a specific element (such as a label or function name) does not require any arguments. This symbol also anticipates future enhancements, such as a namespace mechanism, that are not yet implemented, reflecting the fact that the MCP framework is still in its preliminary stages.

In the example above, the *input_path* defines the starting directory, while the *output_path* and any user-defined key-value pairs are currently ignored. The application begins execution at the pipeline labeled “main_p”, which serves as the entry point.

This first pipeline contains an initial loop that iterates over the contents of the directory specified by *input_path*. For each item found, the “list_input_dirs_p” pipeline is invoked to print the contents of the directory to standard output using the “print_to_stdout” function. If the item is a subdirectory, an additional loop is triggered to process its contents.

The “processing_files_p” pipeline is responsible for handling the files within these subdirectories. Its current implementation simply prints the file names to standard output as well.

The described pipeline can be executed for instance with the following command line statement:



  	➢	./pipeline_runtime.py
                                   ../mcp_use_case_getting_started/first_use_case.yaml


### Dynamic composition of data pipelines

If an existing YAML data pipeline configuration needs to be adapted to a slightly different use case, an additional YAML configuration can be created containing only the necessary changes. This approach allows for efficient reuse of the original configuration by specifying only incremental modifications or overrides.

For instance, to modify the use case shown in Figure 1, such that it not only prints the names of files in subdirectories but also displays the content of any CSV found, one can define a new YAML configuration that extends the original. In this, you can add new pipelines or override existing ones in the section introduced by the key word *pipeline_extension*.

**Figure 2.**
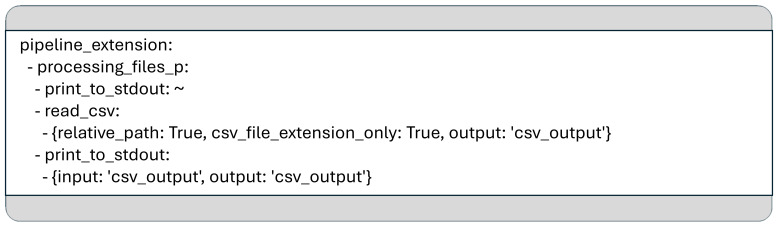
Another YAML configuration which overwrites some parts of the pipeline to display the content of each CSV file located in the subfolders of the given input folder.


Figure 2 shows the content of a YAML file that captures only the modifications which are built on top of the original use case. Since this file relies on the base configuration, it is not standalone and must be used alongside the original YAML configuration. The extended pipeline can be executed with a command such as:

  	➢  ./pipeline_runtime.py
                               ../mcp_use_case_getting_started/first_extension.yaml
                               ../mcp_use_case_getting_started/first_use_case.yaml


In this example, we modified the “processing_files_p” pipeline so that it not only prints the names of directories or files but also outputs the content of any file with a *.csv* extension. To achieve this, we used a generalized Python function called *read_csv*, implemented in the module *mcp_general_functions.mcp_general_io*. This function is designed to meet all the requirements of the MCP framework.

For a detailed explanation of how to develop Python functions for use within MCP pipelines, and how data is passed between functions, refer to Section 4.

### Recursive data pipelines

**Figure 3.**
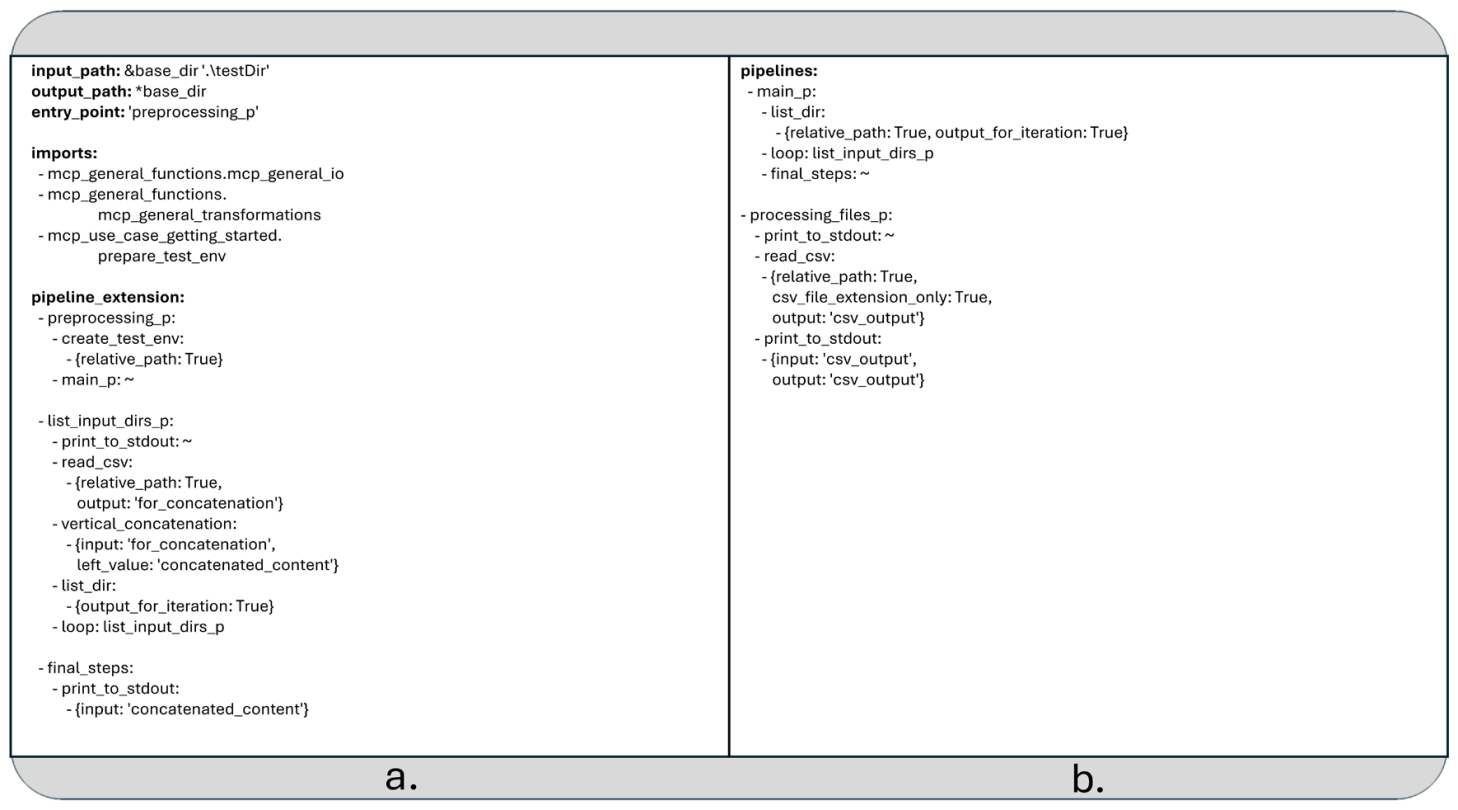
a) The second extension of the data pipeline in YAML format (The recursive part is called “list_input_dirs_p”). b) The rest of the pipeline defined in the previous two YAML files and not overwritten by the latest extension.

MCP supports a limited form of recursion within YAML configuration files. Specifically, recursion is allowed only when a child pipeline is used as a loop kernel (see Figure 3a) and directly or indirectly invokes itself as the kernel of an inner loop. In all other scenarios, such as when a non-loop pipeline simply calls itself, the MCP runtime framework ignores the recursive call and skips its execution.

The recursive loop terminates when there are no more non-empty lists to iterate over, effectively halting further recursive calls.

Unlike the previous two examples, which served primarily as demonstrations, the use case discussed below addresses a more practical scenario: recursively searching for CSV files within a directory structure and concatenating their contents (see Figure 3).

As this example builds upon the combination of the two earlier pipeline configurations, it cannot be executed independently. Figure 3b shows the parts of the pipeline which were defined in the previous two YAML files and are not overwritten by the latest extension. To run the pipeline, you must provide all YAML configuration files together. For example, you can launch the pipeline using the following command:


  	➢  ./pipeline_runtime.py
            ../mcp_use_case_getting_started/second_extension_recursive.yaml
                              ../mcp_use_case_getting_started/first_extension.yaml
                              ../mcp_use_case_getting_started/first_use_case.yaml



The data pipeline depicted on Figure 3 utilizes two additional python functions:

- *create_test_env*, located in the module *mcp_use_case_getting_started.prepare_test_env*, creates a five-level-deep directory structure under the specified base directory (if it doesn't already exist) and distributes some generated CSV files throughout it.
- *vertical_concatenation* found in *mcp_general_functions.mcp_general_transformations*, appends a given matrix to an existing Pandas DataFrame.

The recursive logic is implemented within the pipeline “list_input_dirs_p”, which serves as a loop kernel. This element is directly self-referencing and is invoked from another pipeline called “main_p *”.* The latter one initially generates a list of entries from the directory defined in the YAML field *input_path* and applies “list_input_dirs_p” to each entry.

The “list_input_dirs_p” pipeline prints the name of each directory or file to the standard output. If the entry is a CSV file, its contents are read and stored under the label “for_concatenation”. These contents are then passed to the *vertical_concatenation* function, which appends them to a cumulative data structure labeled “concatenated_content”.

If the entry is a directory, a new list is created from its contents, and “list_input_dirs_p” is recursively called on each element in that list.

Finally, the pipeline element “final_steps” prints the fully concatenated result referenced by the label “concatenated_content” to the standard output. Although the pipeline element “processing_files_p” given previously is still included in the configuration, it is not part of the current execution chain and remains unused in this example.

### Operation

The execution of a set of data transformations using the configuration described above is straightforward and does not rely on a specific runtime environment. The MCP tool requires Python 3 and can be executed like any standard Python program. For dependency management, we use Poetry [ Eustace *et al.*, 2023], which simplifies the setup and maintenance of the development environment.

Core data transformation operations as well as input/output handling are primarily built on the functionality provided by the Pandas library. During the initial development phase, our focus was on implementing the core concept and validating it through three distinct use cases, as discussed in Section 5.

In our test cases, the MCP tool is invoked via the command line with one or more YAML configuration files as arguments:


    1.  pipeline_runtime.py use_case_changes_n.yaml ... use_case_changes_1.yaml basic_use_case.yaml


When multiple configuration files are provided, they are merged into a single cohesive configuration using the Lasagna tool [ RISC\Lasagna, 2021]. Lasagna layers configurations on top of one another, supporting overrides and extensions, and enhances clarity in more complex setups. It also works with general YAML files and improves understandability in the case of more complex configurations.

**Figure 4.**
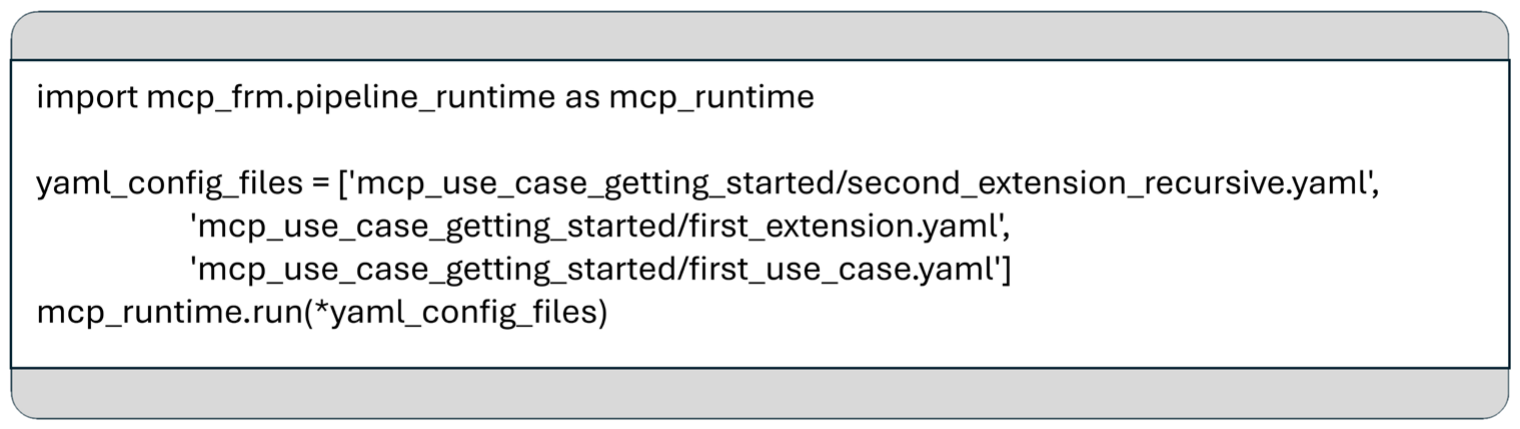
Triggering the execution of MCP pipeline from Python.

Moreover, the MCP framework can be used programmatically as a standard Python library, enabling pipeline executions directly from source code (see Figure 4). This flexibility allows seamless integration into larger software systems and simplifies the refactoring of existing codebases.

## 4. Requirements and features

This section addresses two essential aspects: the requirements that Python functions must fulfill to be referenced within an MCP YAML data pipeline, and the built-in functionality provided by the MCP framework.

### Requirements

**Figure 5.**
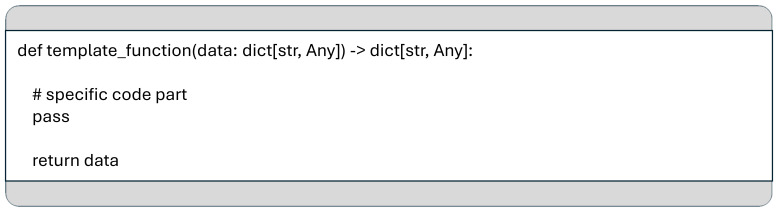
A template for functions used in the data pipeline, including the function signature and return value as required by the MCP framework.

Data is passed between consecutive functions in a pipeline using a Python dictionary, where keys are strings and values can be of any type, see Figure 5.

The dictionary keys serve as labels that identify the data structures passed between functions. The value types vary depending on the requirements of each processing step and can range from simple data types to complex structures, such as Pandas DataFrames, which commonly passed from a data ingestion stage to a processing stage. This approach ensures that data transformation functions maintain a consistent signature across various use cases, enhancing their reusability.

### Key/value pairs and function parameters given in the YAML configuration

When an MCP program starts, the framework first parses non-pipeline-specific information, such as key-value pairs, database configurations, and input/output directory paths, from the provided configuration files. This information is then stored in the aforementioned dictionary under the predefined label “meta” as a JSON-formatted string, ensuring it is easily accessible to all functions throughout the pipeline.

In addition to passing data between functions, the MCP framework also allows extra arguments to be specified directly within the YAML pipeline definition. These parameters, defined for individual function calls, are temporarily added to the “meta” JSON object by the MCP runtime framework just before the corresponding function is executed.

**Figure 6.**
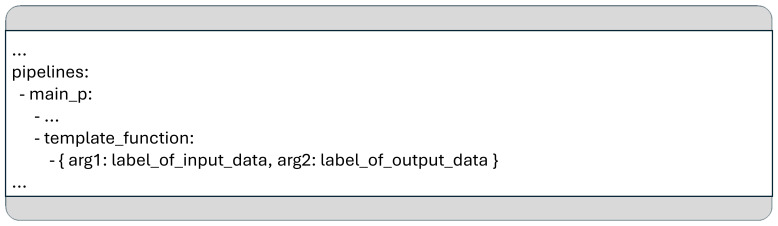
An example for the syntax to specify additional arguments for a function in the YAML configuration.

For example, the function named “template_function” in Figure 6 includes two additional arguments: “arg1” and “arg2”. Such arguments can be used to pass either specific values (e.g.: hyperparameters) or string labels that identify (input/output) data structures within the dictionary passed to the function.

**Figure 7.**
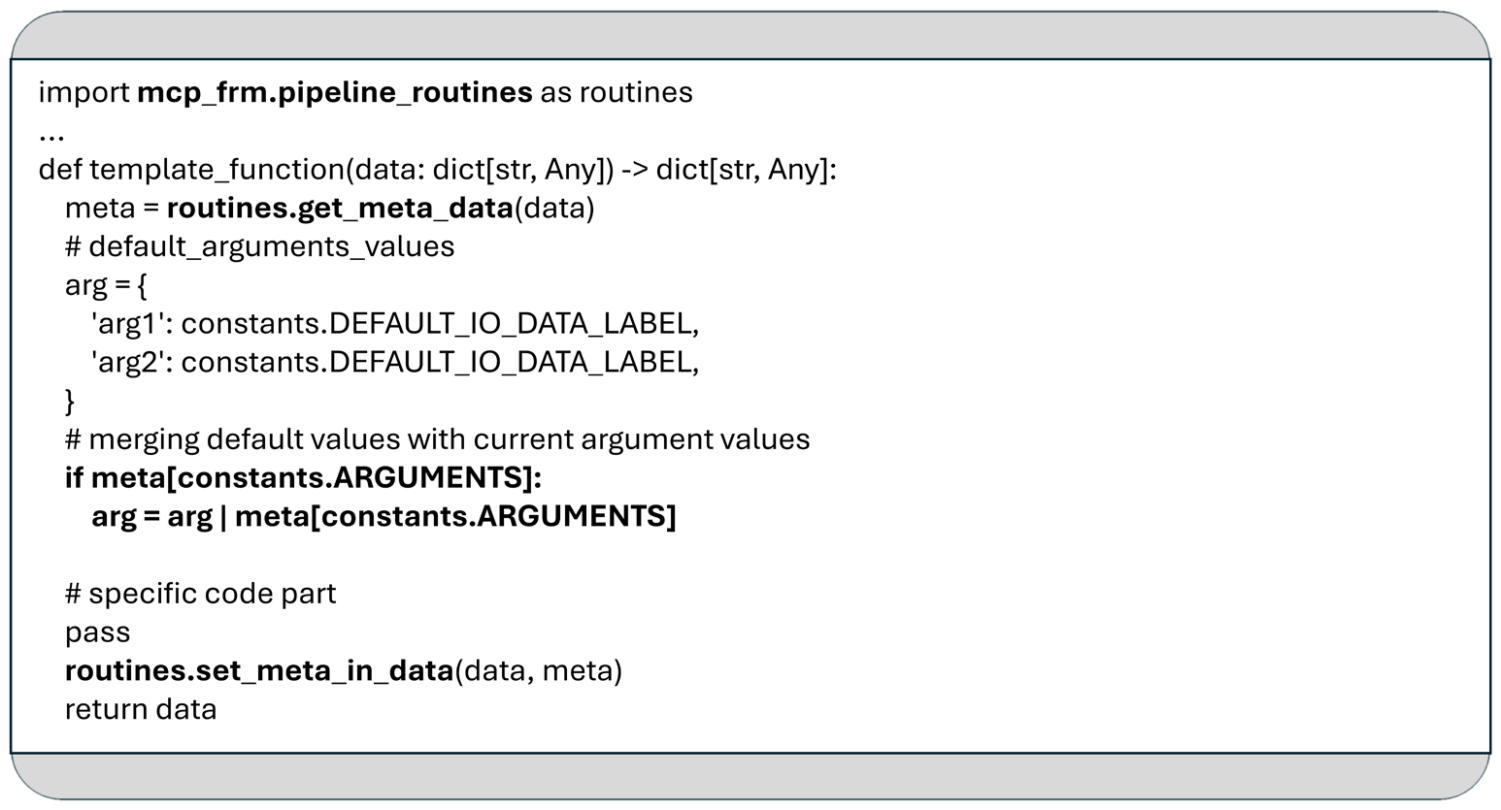
The example demonstrates the use of the functions *get_meta_data* and *set_meta_in_data*, as well as the process for retrieving argument values from the JSON object "meta" as defined in the YAML configuration.

As shown in Figure 7, these function-specific arguments are accessible via the “arg” field within the meta string, which is stored as a JSON object. This “arg” field is updated before each function call to reflect the parameters defined in the YAML configuration. Two core utility functions facilitate this mechanism:

- *get_meta_data:* This function deserializes the content of the JSON string “meta” and is responsible for passing the function parameters specified in the YAML configuration.
- *set_meta_in_data*: This function Serializes and updates the meta string within the dictionary that will be returned at the end of the function execution, ensuring any changes to the metadata persist for subsequent steps.

It is recommended to define default values for all expected arguments within the implementation of any function used in an MCP data pipeline. This allows functions to operate reliably even when specific parameters are not explicitly provided in the YAML configuration.

For example, in Figure 7, the function uses the same default label (defined in “constants.DEFAULT_IO_DATA_LABEL”) to identify both its input and output within the shared data dictionary. As a recommended best practice, each MCP pipeline function should use the same label for input and output by default, enabling seamless handoff of data between consecutive functions without requiring explicit configuration. These default values are initially stored in the “arg” dictionary and can be overridden by user-defined values provided in the YAML configuration, as shown in Figure 6.

### Loops

A common use case in data processing is the need to apply the same code to every element in a dataset. To support this pattern, the MCP framework includes loop functionality, which allows a defined segment of the pipeline in the YAML configuration to be executed iteratively over the elements of a specified list.

Loops are declared using the loop keyword. As illustrated in Figure 1, MCP supports nested loop structures: the outer loop executes the pipeline labeled “list_subdirs_p”, while the inner loop is associated with the “processing_files_p” pipeline.

**Figure 8.**
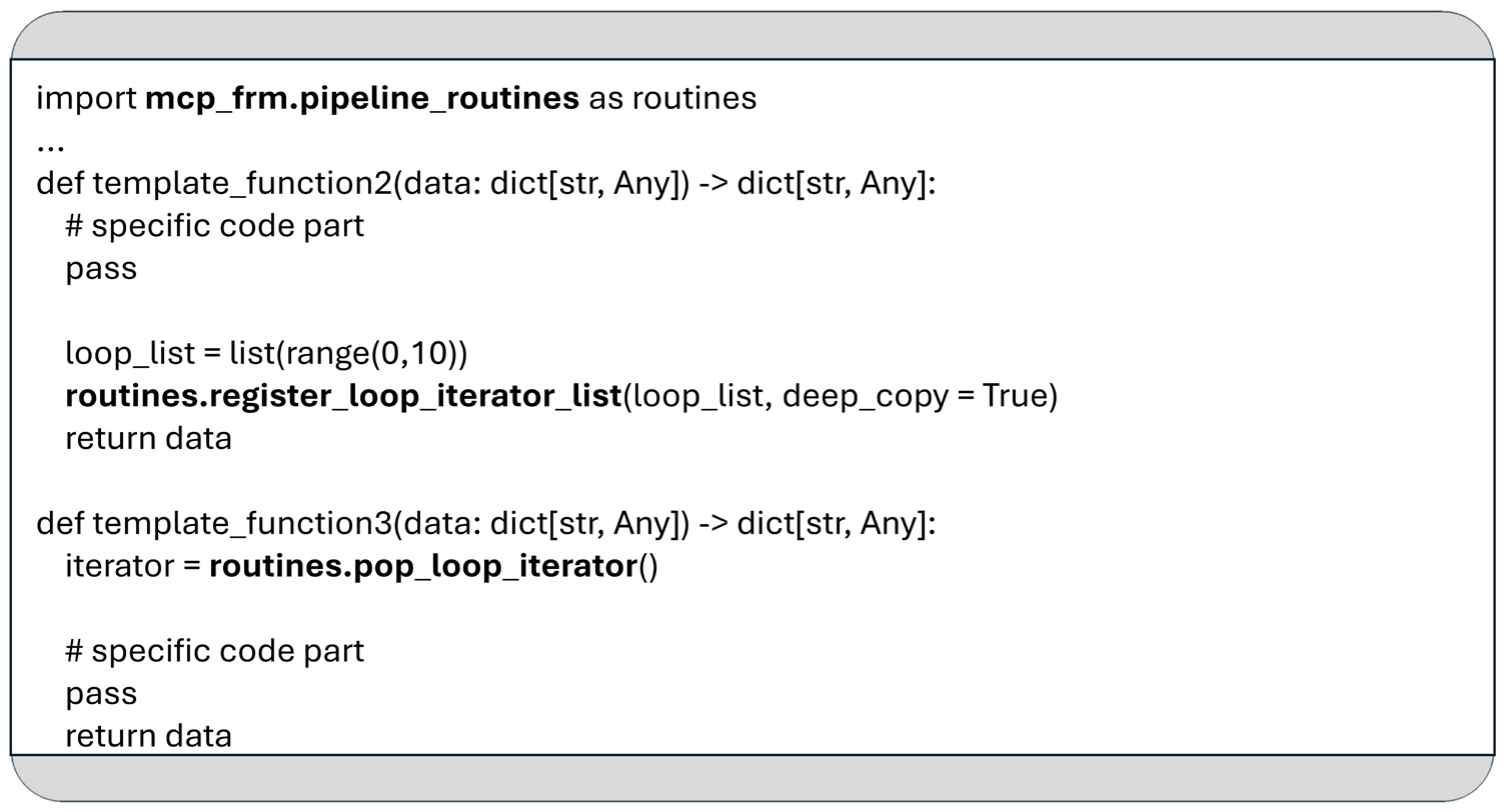
An example to demonstrate the usage of the functions *register_loop_iterator_list* and *pop_loop_iterator*.

Before a loop can be executed, the list to be iterated over must be registered using the *register_loop_iterator_list* function. This is demonstrated in the implementation of template_function2 shown in Figure 8. Once one or more lists are registered, MCP automatically applies the defined loop body to each element of the upcoming list when execution reaches the corresponding loop keyword in the YAML configuration.

If no list is provided for the upcoming loop, the statements inside the loop will not be executed. The current list element to be processed by the loop can be accessed with the *pop_loop_iterator* function, as shown in Figure 8.

## 5. Results

Using configuration files to avoid the repeated implementation of similar code segments is conceptually like refactoring logic into separate functions or modules. However, configuration-based design offers greater flexibility, particularly compared to the constraints of imperative programming languages like Python, where the number of parameters and return values a function can handle is practically limited. Configurations can handle arbitrary sets of inputs/outputs without the complexity or limitations of writing and managing highly parameterized functions directly in code.

The long-term advantages of this approach become more pronounced as the system evolves. As more and more generalized modules and functions are developed, they can reduce duplication and improve maintainability without sacrificing flexibility. Over time, this growing library of components allows developers to construct increasingly complex workflows primarily through the composition and customization of configuration files.

To evaluate the effectiveness of the MCP framework in its current state, we focused on the reduction in development effort compared to manually implementing similar data transformation logic. For this assessment, we analyzed three real-world use cases, which originate from ongoing research projects. Since their original implementations were created without MCP, they provide a natural baseline for assessing how much effort and thus cost can be saved by adopting the MCP approach.

Comparing implementation efforts is a topic that has been the focus of much research. An example is the cyclomatic complexity [ McCabe, 1976], which focuses on the number of alternative paths an execution can take through a program. We are relying on that for our initial evaluation of program complexity reduction introduced by MCP.

Given that the current MCP framework includes only a limited set of generalized functions (momentarily around a few dozen only) we intentionally selected relatively simple problems for this assessment. These allow us to more clearly demonstrate the potential benefits of function reuse within the framework.

Before comparing the cyclomatic complexity of the different implementations for the three use cases, it is important to outline that the development of generalized functions is inherently more complex than use-case-specific code.

- A part of this complexity stems from various boilerplate codes, some of which can be hidden from developers using techniques such as Python decorators.
- Another part of it comes from the broader problem coverage and more sophisticated logic required to make these functions reusable in multiple contexts.

As a result, the implementation of a use case, assuming all functions are written from scratch, may cause a higher challenge in MCP. However, once a so-called general-purpose function is created, it can be reused multiple times without modification.

In such cases, when solving a later problem, the effort required to implement it and its complexity no longer needs to be considered.

To capture this distinction, we calculated **two cyclomatic complexity values** for each MCP-based solution (refer to Table 1 through Table 6):

- **Use-case-specific complexity only** – This metric includes only the complexity of the code written specifically for the use case, excluding the reusable general-purpose functions (indicated with red figures).
- **Full complexity including reusable functions** – This value includes the total complexity of the solution, assuming that all reusable functions were implemented from scratch within that use case (shown in black numbers).

The cyclomatic complexity for all implementations was calculated using the Python tool **Radon** [ Radon, 2024].

### Universidad Pable Olavide (UPO) - Solar cell quantum efficiency measurements

Within the scope of the Platform Zero project ( https://www.platform-zero-project.eu/) the Universidad Pablo Olavide was supported in extracting features from their raw measurement data and to convert them into a defined intermediate text format based on CSV. This format will subsequently be used to exchange the extracted features within the project consortium for further machine learning training. This data preprocessing and conversion includes a series of computations as well as numerically integrating the output of a solar cell in terms of the electrical current across different wavelengths of light to compute quantum efficiency.

Comparing the cyclomatic complexity between the initial implementation and the MCP approach gives the following results, see Table 1 and Table 2.

**Table 1.** Quantum efficiency data integration – cyclomatic complexity of basic implementation.

Function	Complexity
Main	2
calc_write_sol1	5
calc_write_sol2	4
interpolate_help	5
csv_concatenation_vertical	4
calculate_integral_current_density	3
calculate_eqe_perc	6
calculate_electron_flux	4
write_EQE_sol1	7
write_EQE_sol2	5
**Sum**	**45**

**Table 2.** Quantum efficiency data integration – cyclomatic complexity of MCP implementation.

Function	Complexity
read_csv	10
interpolate_first_column	7
vertical_concatenation	8
init_calc_eqe_perc	2
init_calc_integral_current_density	2
calculate_integral_current_density	3
calculate_eqe_perc	6
calculate_electron_flux	4
compose_output	3
write_eqe_sol1	7
write_eqe_sol2	5
**Sum**	**57 (33)**

Interpreting these results, it can be said that the basic implementation uses the Pandas library call for reading CSV files. Table 1. contains the functions “calc_write_sol1” and “calc_write_sol2”, which perform some basic initialization and load some csv files containing numerous constants values for the calculation. Within the MCP solution, all is done in the YAML data pipeline configuration through definition of key value pairs and by calling the generalized function called “read_csv” with different parameters in the YAML data pipeline configuration.

### Fronius – Industrial welding

Within the scope of the Metafacturing project ( https://metafacturing.eu/) a solution to resample and merge the data from an industrial welding process has been developed. The raw data has been provided in different sampling rates – 1 KHz and 10 KHz respectively – and thus needed to be partially up-sampled and merged. Subsequently, the start and end timestamps for a weld seam have also been stored as additional meta-information.

Comparing the cyclomatic complexity between the initial implementation and the MCP approach gives the following results, see Table 3 and Table 4.

**Table 3.** Welding data resampling and merging – cyclomatic complexity of basic implementation.

Function	Complexity
Main	2
Intro	3
Unzip	2
csv_concatenation	3
resample_df	4
read_all_csv	5
resample_df	3
check_sample_rate_quantile	1
add_columns_to_df	2
**Sum**	**25**

**Table 4.** Welding data resampling and merging – cyclomatic complexity of MCP implementation.

Function	Complexity
determine_end_date_from_filename_v2	2
determine_output_filename	1
Unzip	9
read_csv	10
write_parquet	6
merging_columns	4
resample_df	3
check_sample_rate_quantile	1
**Sum**	**38** (9)

Here again for reading CSV files a custom method has been used in the MCP implementation as compared to the Pandas function in the base implementation, which increases the cyclomatic complexity by six.

### LKR (Leichtmetallkompetenzzentrum Ranshofen) – Simulation of aluminium casting

Within the scope of the opt1mus project ( https://projekte.ffg.at/projekt/4641718) a data importer to ingest tabular text data into InfluxDB - a time-series database - has been developed. The focus has been put on parsing the structured text format of the source file and injecting the data into the time series database efficiently. The data itself represents the output of a simulation run of horizontal aluminum die casting. It is provided in a structured text format as input, parsed into a Pandas dataframe and then subsequently stored into InfluxDB.

Comparing the cyclomatic complexity between the initial implementation and the MCP approach gives the following results, see Table 5 and Table 6.

**Table 5.** Data transformation and storing – cyclomatic complexity of basic implementation.

Function	Complexity
Main	2
simulation_data_use_case	3
read_simulation_data_from_csv	6
write_dataframe_list_to_db	2
**Sum**	**13**

**Table 6.** Data transformation and storing – cyclomatic complexity of MCP implementation.

Function	Complexity
get_simulation_id	2
data_conversions	2
read_csv	10
influx_df_write	2
**Sum**	**16 (4)**

### Lesson learned

In summary, cyclomatic complexity was used as a metric to compare the implementation complexity of traditional Python-based solutions against those built using the MCP framework. The results across all evaluated scenarios suggest the following:

- **Initial setup overhead:** Developing MCP-based solutions requires a higher initial effort if no reusable function libraries are available. This highlights that the approach outlined in this paper depends on an upfront investment either by a team or community to build and share a library of common components before its full benefits can be realized.
- **Long-term efficiency:** Once a set of generalized functions is in place, which covers typical data engineering tasks (such as common transformations or interactions with different file formats and storage systems), the MCP-based implementations show a modest reduction in cyclomatic complexity. This demonstrates the potential of the framework to simplify and accelerate the development of diverse data engineering pipelines over time.

While function reuse is not exclusive to MCP, our experience suggests that the MCP approach inherently promotes code reuse. By starting with a configuration file and focusing only on truly new code components, developers are less likely to reimplement existing code. In contrast, directly starting with Python code often encourages redundant implementations, as such tasks can appear deceptively simple.

This emphasis on reuse provides a comparable advantage to low-code platforms like NiFi, with the added benefit of seamless integration for custom code components.

## 6. Conclusions

This paper presents a lightweight and flexible methodology for automatically constructing data pipelines from YAML-based configuration files. The primary goal is to reduce repetitive code while preserving full flexibility for integrating custom Python modules. We outlined the core concepts of the approach, detailed the structure of the configuration files, and demonstrated how it simplifies the implementation of ETL workflows. Additionally, we discussed its impact on reducing cyclomatic complexity when compared to traditional, hand-coded implementations.

The proposed solution is actively utilized by our team to streamline the effort and reduce the cost associated with developing real-life data engineering use cases as well as continuously improving and extending it to meet evolving demands.

### Airflow integration

As mentioned earlier, we have considered the integration of MCP with Apache Airflow, especially to support real-world data engineering scenarios. In such a setup, Airflow DAGs (Directed Acyclic Graphs) typically manage when and under what conditions an MCP data pipeline should be executed. For instance, an MCP pipeline might be triggered only when new data becomes available either in the file system or in a source database indicating the need for processing.

This approach enables seamless automation, where Airflow handles scheduling, dependency resolution, and monitoring, while MCP is responsible for the core data transformation logic. Since the latter is specified through a combination of some general-purpose Python functions and lightweight, YAML-based data pipeline specifications, making it easy to develop, extend, and maintain processes with minimal code duplication.

The simplest and most direct method to invoke an MCP pipeline from within an Airflow DAG is by using the *BashOperator*. This approach allows the MCP framework to be launched as a standard command-line process, making integration straightforward and requiring minimal setup.

For more advanced scenarios, the integration could also include Airflow’s sensing mechanisms (e.g., *FileSensor, TimeSensor, ExternalTaskSensor, or TriggerDagRunOperator*) to check for specific trigger conditions, such as the presence of a file or the successful completion of an upstream task, before initiating the MCP workflow. Additionally, for isolated and reproducible deployments, the *DockerOperato*r can be employed to launch MCP within a dedicated Docker container. This encapsulates the entire runtime environment, making the execution environment consistent across different systems and eliminating dependencies on the host system.

This hybrid model leverages Airflow’s strengths in orchestration and monitoring, while MCP provides flexibility and reusability with its lightweight, configuration-driven data pipelines.

### Future work

As part of our ongoing efforts to enhance developer experience and streamline data pipeline development, we have recently improved the usability of the MCP framework:

- A Python decorator for handling default parameters for pipeline functions. It abstracts away common boilerplate tasks, such as accessing loop iterators and extracting function-specific arguments from the “meta” JSON string, etc., into a clean, reusable layer.
- Using code injection to access parameters more conveniently from inside of a pipeline function.

By automating these repetitive patterns, we intend to simplify function implementation, reduce the likelihood of errors, and promote cleaner, more maintainable code across our MCP-based data engineering projects.

Continuing this work, we will increase the number of pre-implemented functions as well as the usability of the overall framework.

## Ethics and consent

Ethical approval and consent were not required.

## Data Availability

No data associated with the article. **Source code available from:** https://github.com/kbosa-risc/mcpf-getting-started **Archived software available from:** https://doi.org/10.5281/zenodo.15975472 **License:** MIT License ***How to start*** If one would like to try out the MCP framework, a possible starting point would be our getting started example available on https://github.com/kbosa-risc/mcpf-getting-started This repository includes the source files for the three examples presented, along with configuration files (pyproject.toml and poetry.lock) for installing the necessary dependencies of the publicly available MCP framework using *Poetry,* a dependency management and packaging tool for Python. ***Prerequisites*** Python 3.10 or later ***Set up the project*** Install prerequisites for virtual environments. You have to do this only once per computer (linux): ➢  sudo apt-get -y update && sudo apt-get -y install python3 python3-venv basic_use_case.yaml Unzip the downloaded file. Move to the extracted dirtectory, e.g.: ➢  cd mcpf-getting-startedcd mcpf-getting-started This project uses `poetry` as a build system. It is recommended to install poetry in a virtual environment (linux): ➢  python3 -m venv ../mcpf-getting-started-venv ➢  ( . ../mcpf-getting-started-venv/bin/activate; pip3 install poetry==1.8.5 ) Now make available the executables of the virtual environment. You have to do this every time when you start a new console session or add the commands to your shell startup script (e.g. `$HOME/.bashrc` under linux): ➢  export PATH=$(pwd)/../mcpf-getting-started-venv/bin:$PATH ➢  hash -r 2> /dev/null To shut down the virtual environment, simply close the console. ***Install dependencies*** The Minimalist Configurable Pipeline framework is built on a modular stack of pipeline functions. Many pipeline functions are provided by add-on packages: mcp[core]: Core functionality of the mcpf framework. mcpf[io]: Basic input/output functions like listing directories. mcpf[postgres]: Postgres-related database functions. mcpf[xform]: Versatile transformation functions. For running the examples, we only need to install mcpf[io] and mcpf[xform] as specified in the file pyproject.toml (the core will be installed as a required dependency): ➢ poetry install ***Overview*** The mcpf-getting-started project consists of three pipeline configurations as discussed in Section 3: first_use_case.yaml is the base configuration, first_extension.yaml and second_extension_recursive.yaml are extension configurations inherited from first_use_case.yaml. ***How to execute examples*** Assuming you are in the root of the working directory of mcpf-getting-started (linux): ➢ poetry run python3 -m mcpf_core.run getting_started/first_use_case.yaml
   ➢ poetry run python3 -m mcpf_core.run \
				 getting_started/first_extension.yaml \
				 getting_started/first_use_case.yaml
   ➢ poetry run python3 -m mcpf_core.run \
				 getting_started/second_extension_recursive.yaml \
				 getting_started/first_extension.yaml \
				 getting_started/first_use_case.yaml We have reviewed the reporting guidelines provided by the EQUATOR Network and FAIRSharing. No specific reporting guideline was found that applies to the type of research presented in this manuscript."
