# Supplementary material for: Automated Execution of Data Pipelines based on Configuration Files
Source: Open Res Eur. 2026 Apr 2;5:291. Originally published 2025 Sep 19. [Version 2] doi: 10.12688/openreseurope.21019.2 (PMC12784049; doi:10.12688/openreseurope.21019.2)
Supplement: Peer review history

# Reviewer report: Reviewer response for version 1

**DOI:** 10.21956/openreseurope.22738.r63864

**Published:** 2026-01-08

**Authors:** Uta Störl, Valerie Restat

**Affiliations:** 1 FernUniversitat in Hagen Fakultat fur Mathematik und Informatik (Ringgold ID: 229926), Hagen, North Rhine-Westphalia, Germany

This paper introduces the Minimalist Configurable Pipeline (MPC) framework, a software for the automated execution of python data pipelines using configuration files to simplify code reusability and increase maintainability. The paper is well written and easy to understand. It deals with a very relevant topic. The associated software is provided, and its application is explained through various use cases.

 However, the following aspects could still be improved:

 - **Lack of classification of applicability:** The authors specifically address their use cases without going into detail about the context of ETL processes and their special characteristics. It remains unclear for which scenarios MCP is suitable and for which it is not. In addition, other tools are described in the related work section, but greater emphasis should be placed on how MCP differs from high-level tools, for example, and where the respective advantages and disadvantages lie in their use.

**- Lack of discussion of the results:** For the evaluation, the cyclomatic complexity of a basic implementation is compared with the MCP implementation for three use cases. However, there is no explanation for why cyclomatic complexity was chosen and what alternatives there might have been. Moreover, there is no in-depth discussion and interpretation of the results. It could also be discussed in more detail how the use of MCP differs from parameterized code. The strengths of MCP should be emphasized more here.

 Although the article is written in a very understandable way, certain questions remain unanswered:

**- Parallelism:** It remains unclear how to specify in the configuration file which steps of a pipeline can be executed in parallel.

**- YAML vs. JSON:** It remains unclear why YAML was chosen for the configuration files. Especially since meta information is transferred in JSON format later on, the question arises as to why a uniform format was not chosen.

**- Parameters of python functions:** Python functions offer many different parameters. For example, a delimiter can be specified for pandas read_csv(). Can such parameters also be set via the configuration files, or is redundant code required for this?

**- Handling different software versions:** How is it handled when different use cases use different versions of software (e.g., pandas version)? Is a common code base a mandatory requirement for the applicability of the configuration files?

**- Airflow in Conclusion: **Most of the conclusion consists of a description of Airflow integration. This should rather be a separate section, with the conclusion only summarizing the article and providing an outlook on future work

**- Missing figure showing the overall architecture:** It would be helpful for understanding if there were a figure showing how the configuration files and custom code interact. This would make it easier to understand which aspects of the code can be referenced in the configuration files.

**Minor comments:**

 - Incorrect capitalization on page 3 ("Key steps")

 - Some statements with missing references

 - The Git repo is very comprehensive and well described, but some links (e.g., in the mfcp-core package) lead to an internal Gitlab that is not publicly accessible.

Is the rationale for developing the new software tool clearly explained?

Yes

Is the description of the software tool technically sound?

Yes

Are sufficient details of the code, methods and analysis (if applicable) provided to allow replication of the software development and its use by others?

Partly

Is sufficient information provided to allow interpretation of the expected output datasets and any results generated using the tool?

Partly

Reviewer Expertise:

Data Management, Data Engineering, Data Cleaning

We confirm that we have read this submission and believe that we have an appropriate level of expertise to confirm that it is of an acceptable scientific standard.

# Reviewer report: Reviewer response for version 1

**DOI:** 10.21956/openreseurope.22738.r61684

**Published:** 2025-11-11

**Authors:** Dr. Virendra Kumar Tiwari

**Affiliations:** 1 Lakshmi Narain College of Technology, Bhopal, Madhya Pradesh, India

This software tool article introduces the Minimalist Configurable Pipeline (MCP) framework, a lightweight, configuration-driven tool for automating the execution of data pipelines in Python. The authors, Károly Bósa and Paul Heinzlreiter from RISC Software GmbH, argue that data preparation tasks (e.g., ETL/ELT processes for machine learning, visualization, or reporting) often involve repetitive code snippets despite using efficient libraries like Pandas and Dask. MCP addresses this by allowing users to define pipelines declaratively in YAML configuration files, which reference reusable Python functions or modules. This shifts focus from rewriting code to configuring and reusing modular components, improving maintainability, reducing duplication, and supporting basic control structures like loops.

 The article is scientifically sound, well-written, and aligns with the standards for a software tool paper in Open Research Europe. It provides a balanced, evidence-based contribution to data engineering, with practical value for Python developers in ETL workflows. All questions are answered "Yes," so no major revisions are required for soundness. However, minor enhancements could improve clarity and impact (these are suggestions, not mandatory):

- **Expand on GitHub Repo Usage**: While the repo is linked, briefly mention in the methods what it contains (e.g., sample configs, core modules) to guide readers.
- **Include Figure Captions/Visuals**: The YAML example (Figure 1) is referenced but not shown in the text; ensure visuals are embedded or described fully.
- **Broader Evaluation**: Section 5 focuses on cyclomatic complexity; adding runtime performance or user feedback metrics could strengthen results, though the current analysis is sufficient.
- **Accessibility**: Confirm the GitHub repo includes documentation (e.g., README with installation/setup), as this supports replication.

Is the rationale for developing the new software tool clearly explained?

Yes

Is the description of the software tool technically sound?

Yes

Are sufficient details of the code, methods and analysis (if applicable) provided to allow replication of the software development and its use by others?

Yes

Is sufficient information provided to allow interpretation of the expected output datasets and any results generated using the tool?

Yes

Reviewer Expertise:

AI, ML ,Cloud etc

I confirm that I have read this submission and believe that I have an appropriate level of expertise to confirm that it is of an acceptable scientific standard.
